# In Silico and In Vitro Screening and Mechanisms of Angiotensin I-Converting Enzyme Inhibitory Peptides from Protein Hydrolysates of Royal Jelly

**DOI:** 10.3390/foods15091536

**Published:** 2026-04-29

**Authors:** Ying Zhang, Shipeng Guo, Haoxiang Miao, Yafei Gu, Jian Zhang

**Affiliations:** 1 College of Food Science and Engineering, Tianjin University of Science and Technology, Tianjin 300457, China; zhangying@tust.edu.cn; 2 College of Biotechnology, Tianjin University of Science and Technology, Tianjin 300457, China; gsp1054743549@163.com (S.G.); mhx990321@163.com (H.M.); 13934965657@163.com (Y.G.)

**Keywords:** royal jelly, ACE inhibitory peptide, in silico, purification, molecular docking

## Abstract

This work focused on the identification of angiotensin I-converting enzyme (ACE) inhibitory peptides from royal jelly (RJ) proteins and elucidated their inhibition patterns and mechanisms. RJ proteins were analyzed for ACE inhibition potential using in silico tools, and suitable enzymes were selected for peptide release. Hydrolysis conditions were optimized using response surface methodology (RSM), and the resulting peptides were fractionated and purified. Mass spectrometry identified 57 peptides, with seven selected for synthesis based on scoring. IDFDF, DVNFR, and SFHRL showed the highest ACE inhibition, with IC_50_ values of 16.9 μM, 42.5 μM, and 242.6 μM, respectively. Lineweaver–Burk plots revealed IDFDF as a competitive inhibitor, DVNFR as a non-competitive inhibitor, and SFHRL as a mixed inhibitor. Molecular docking indicated that peptide–ACE interactions were primarily mediated through hydrogen bonds and Zn(II) coordination. This work promotes the sustainable utilization of RJ and the development of ACE inhibitory peptides derived from food sources.

## 1. Introduction

Royal jelly (RJ) is a natural substance secreted by the hypopharyngeal and mandibular glands of nurse honeybees (*Apis mellifera*) [1]. It plays a crucial role in the development of queen bees and larvae within honeybee colonies and has been broadly adopted in health-promoting and beauty-enhancing products for humans [2]. The caste system in honeybees is nutritionally mediated, with larvae fed exclusively on RJ developing into queens [3,4]. Notably, queen honeybees exhibit a body size approximately double and a lifespan tenfold greater than that of worker bees of the same genotype [2].

RJ has been shown to possess diverse bioactivities, including antioxidant, anti-inflammatory, anti-aging, neuroprotective, antimicrobial, anti-allergic, and antitumoral properties [5,6]. However, several limitations hinder its commercial potential, including poor stability, limited water solubility, an undesirable spicy and sour taste, and the potential to cause allergic reactions in susceptible individuals [7]. China is the world’s largest producer and exporter of RJ, with an annual production exceeding 4000 tons and a market value surpassing $2.5 billion, accounting for approximately 90% of global RJ production [8,9]. However, a significant portion of RJ is exported in raw form, resulting in relatively low profitability. Therefore, extensive processing of RJ is essential to address these issues.

RJ consists of approximately 60–70% water, 9–18% proteins, 11–23% carbohydrates, and 4–8% lipids [10]. It also contains trace amounts of mineral salts, phenols, flavonoids, free amino acids, vitamins, and other components. The protein fraction of RJ is dominated by MRJPs, which represent 83–90% of the total proteins [10]; MRJP1 is the most abundant among them, making up 31–66% of total RJ proteins, while MRJP3, MRJP2, and MRJP5 account for 26%, 16%, and 9%, respectively [11].

As the primary bioactive component of RJ, RJ proteins are widely used in nutraceuticals, cosmetics, and functional foods due to their antioxidant, anti-inflammatory, and immunomodulatory properties. In nutraceuticals, they are formulated into oral supplements for anti-aging and metabolic regulation; in cosmetics, they are incorporated into skin-care products to promote skin cell proliferation, providing moisturizing and anti-wrinkle benefits. However, the low bioavailability and unexploited latent bioactivities of native RJ proteins limit their further industrial application. To overcome these bottlenecks, enzymatic hydrolysis to produce bioactive peptides represents a key strategy.

Bioactive peptides (2–20 amino acids) are inactive within parent proteins but become functional upon release [12]. Despite this potential, RJ-derived bioactive peptides remain underexplored, with only a few studies reporting antioxidant, ACE inhibitory, and renin inhibitory activities [13,14,15,16]. ACE inhibitory peptides are particularly valuable for lowering blood pressure and reducing hypertension-related risks [17]. Unlike synthetic ACE inhibitors (e.g., captopril), which often cause adverse effects [18], natural ACE inhibitory peptides offer a safer alternative. However, only a limited number of such peptides (e.g., Ile-Tyr, Val-Tyr, and Lys-Phe) have been identified from RJ [14,15], and their mechanisms of action remain poorly understood.

Selecting appropriate proteases and optimizing hydrolysis conditions can enrich hydrolysates with ACE inhibitory activity. Such hydrolysates can be directly used as industrial products, addressing the application bottlenecks of RJ proteins. Further isolation and purification to obtain ACE inhibitory peptides with defined amino acid sequences would enable large-scale chemical synthesis, followed by mechanistic studies and in vivo functional evaluations, thereby laying the foundation for ACE inhibitory drug development.

This study was designed to: (a) apply in silico tools to screen for appropriate proteases capable of producing ACE inhibitory peptides from RJ proteins; (b) optimize the enzymatic hydrolysis conditions using response surface methodology; (c) fractionate and purify the ACE inhibitory peptides by successive chromatography on Sephadex G-25, anion exchange, and RP-HPLC; (d) identify the peptide sequences in the most active fractions via LC-MS/MS; (e) synthesize the most promising peptides and measure their IC_50_ values; and (f) investigate their inhibition mechanisms against ACE.

## 2. Materials and Methods

### 2.1. Materials and Reagents

The RJ proteins were supplied by Beijing Jinwang Health Technology Co., Ltd. (Beijing, China), with a protein purity of 85% on a dry weight basis and residual components of carbohydrates (8.0%), lipids (3.2%), ash (2.8%), and moisture (1.0%). No exogenous additives were present. Proteinase K (EC 3.4.21.64, 40 U/mg), ACE (from rabbit lung) and its substrate N-[3-(2-Furyl)acryloyl]-L-phenylalanyl-glycyl-glycine (FAPGG) were purchased from Solarbio Science & Technology Co., Ltd. (Beijing, China). Sephadex G-25 was supplied by Shanghai Yuanye BioTechnology Co., Ltd. (Shanghai, China). HPLC-grade acetonitrile, methanol, and trifluoroacetic acid (TFA) were obtained from Fisher Scientific Company (Waltham, MA, USA). All other chemicals used were of analytical reagent grade.

### 2.2. Evaluation of RJ Proteins as a Source of Bioactive Peptides

An in silico analysis was conducted to assess the potential of RJ proteins as a source of bioactive peptides. The sequences of MRJPs used in this study were obtained from the NCBI database. Nine RJ protein sequences were selected, including MRJP1 (AAM73637.1), MRJP2 (ACS66837.1), MRJP3 (ADC55524.1), MRJP4 (ADB82660.1), MRJP5 (NP_001011599.1), MRJP6 (AAQ82184.1), MRJP7 (DAA01512.1), MRJP8 (AAR83734.1), and MRJP9 (AAY21180.1), which collectively account for over 83% of RJ proteins. Using the “PROTEINS”, “ANALYSIS”, and “CALCULATIONS” functions in the BIOPEP-UWM database (http://biochemia.uwm.edu.pl (accessed on 20 March 2025)), the biological activities of MRJPs 1–9 were predicted to explore the potential activities after enzymatic hydrolysis [19].

The frequency of occurrence (A) of peptides with specific biological activities within each MRJP sequence was evaluated using the “calculations” tab. Parameter A was calculated as the proportion of the number of specific bioactive peptides to the total amino acid count in each MRJP sequence, as outlined by Minkiewicz et al. [19]. Additionally, the aggregate occurrence frequency (ΣA) of all bioactive peptides within each protein sequence was assessed.

### 2.3. In Silico Prediction of ACE Inhibitory Peptides from MRJPs

The MRJP sequences were subjected to enzymatic hydrolysis within the BIOPEP-UWM database using the “PROTEINS”, “ANALYSIS”, and “BATCH PROCESSING” features [19]. The analysis included the theoretical degree of hydrolysis (DHt) and the incidence of ACE inhibitory peptides released by proteolytic enzymes. DHt represents the proportion of cleaved peptide bonds to the total number of peptide bonds in a protein sequence [19]. Seven distinct enzymes were used: pepsin (pH 1.3) (EC 3.4.23.1), chymotrypsin (A) (EC 3.4.21.1), trypsin (EC 3.4.21.4), papain (EC 3.4.22.2), stem bromelain (EC 3.4.22.32), subtilisin (EC 3.4.21.62), and proteinase K (EC 3.4.21.64).

### 2.4. Measurement of ACE Inhibitory Activity

The ACE inhibitory activity was determined following the methodology outlined by Cao et al., with slight modifications [20]. Briefly, 10 µL of 0.1 U/mL ACE solution was added to 40 µL of the sample in a 96-well microtiter plate and incubated for 10 min at 37 °C. The reaction was initiated by adding 50 µL of FAPGG buffer (1.0 mmol/L, containing 80 mM HEPES and 0.3 M NaCl, pH 8.3), and the plate was immediately transferred to a multi-wavelength microplate reader set at 37 °C for 30 min. The absorbance values of the sample before and after the reaction were measured at 340 nm and recorded as $b_{1}$ and $b_{2}$. A blank was prepared similarly, replacing the sample with the same volume of 80 mM HEPES buffer. The absorbance difference in the blank was recorded as $a_{1}$ and $a_{2}$. Samples were assayed in triplicate. The ACE inhibitory activity (%) was calculated using the following equation:

$$ACE\;inhibitory\;activity\;(\%)=\left[\left(a_{1}-a_{2}\right)-\left(b_{1}-b_{2}\right)\right]\times 100∕\left(a_{1}-a_{2}\right)$$

The IC_50_, defined as the concentration of inhibitor that suppresses 50% of ACE activity, was determined by plotting the percentage inhibition against peptide concentration.

### 2.5. Optimization of RJ Protease Hydrolysis Process Using Response Surface Methodology

RSM was employed to optimize the enzymatic hydrolysis conditions. The independent variables were enzymatic hydrolysis temperature (A), pH (B), and time (C), with ACE inhibitory activity (Y) as the dependent variable. The Box–Behnken design was used to optimize the processing parameters, with each variable encoded at three levels: −1, 0, and +1 (Table 1).

Data analysis, including model fitting and regression, was performed using Design-Expert software (Version 8.0.6). RJ proteins were dissolved in deionized water, and proteinase K was added at an enzyme-to-substrate (E/S) mass ratio of 1.0%. After adjusting the pH, enzymatic hydrolysis was carried out in a shaking water bath at 100 r/min under the appropriate temperature for a certain period of time. After each hydrolysis step, the hydrolysates were heated at 100 °C for 15 min to inactivate enzymes, cooled to room temperature, and centrifuged at 9600 *g* for 10 min. The resulting supernatants were collected and analyzed for ACE inhibitory activity. The predictive model for enzymatic hydrolysis conditions was validated by assessing the ACE inhibitory potency of the hydrolysate obtained at optimized conditions.

### 2.6. Stepwise Purification of ACE Inhibitory Peptides

The hydrolysate was first filtered through a 0.45 µm polyethersulfone ultrafiltration membrane. The filtrate was then loaded onto a Sephadex G-25 gel column, which was eluted with deionized water at 0.5 mL/min using an SDL-30 protein purification system (Suzhou Sepure Instrument Co., Ltd., Suzhou, China) equipped with a UV detector. The elution was monitored at 214 nm, and fractions were collected at 5 min intervals. The ACE inhibitory activity of each fraction was evaluated, and the protein content was quantified using the Folin phenol method [21]. The most active fraction was further purified on a HiTrap DEAE FF column (16 mm × 100 mm, GE Healthcare, Uppsala, Sweden), pre-equilibrated with 50 mM Tris/HCl buffer (pH 7.0), using the same purification system. Elution was performed with a linear NaCl gradient (0–1 mol/L) in the same buffer. Fractions were collected, desalted, and analyzed for ACE inhibitory activity. Fraction C4 was further purified on a Waters 600 system (equipped with a 2998 PDA detector) using a preparative C18 column (20 × 250 mm, 15 µm, Waters Corporation, Milford, MA, USA). A 150 µL sample (20 mg/mL) was applied and eluted at 1.5 mL/min with a linear gradient of water (solvent A, containing 0.1% TFA) and acetonitrile (solvent B, containing 0.1% TFA). The linear gradient elution program was set as follows: 0–5 min, 0% B (100% A); 5–25 min, 0–50% B; 25–30 min, 50–100% B. This was followed by a 10 min re-equilibration with 100% solvent A. The elution profile was monitored by UV absorbance at 214 nm. The collected subfractions were lyophilized and then evaluated for their ACE inhibitory activity. The most active subfraction was selected for peptide sequence identification.

### 2.7. Peptide Sequence Analysis by LC-MS/MS

Bio-Tech Pack Technology Company Ltd. (Beijing, China) performed LC-MS/MS to identify ACE inhibitory peptides, employing an easy nLC 1200 system (Thermo Fisher Scientific, Waltham, MA, USA) for HPLC separation. Chromatographic separation was achieved using a custom-packed column with Acclaim PepMap RPLC C18 (1.9 µm, 100 Å, 150 µm × 15 cm) media (Dr. Maisch GmbH, Ammerbuch, Germany). The mobile phase was composed of solvent A (0.1% formic acid in water) and solvent B (0.1% formic acid and 80% acetonitrile in water). The flow rate was set at 600 nL/min. The gradient elution profile was as follows: 4% B-phase at 0 min, increasing to 8% at 2 min, 28% at 43 min, 40% at 10 min, 95% at 1 min, and maintained at 95% for 10 min. Mass spectrometry analysis was performed using an Orbitrap Fusion Lumos instrument (Thermo Fisher Scientific, Waltham, MA, USA). The analytical parameters were set as follows: spray voltage of 2.2 kV, capillary temperature of 270 °C, resolution of 70,000 at 400 *m*/*z* for the primary mass analyzer, and 17,500 at *m*/*z* 200 for the secondary analyzer. The scan range for precursor ions was *m*/*z* 100–1500. The automatic gain control (AGC) target for MS1 was 3 × 10^6^ with an ion injection time of 100 ms, while for MS2, the AGC target was 1 × 10^5^ with an ion injection time of 50 ms. The ion selection window was 1.6 *m*/*z*, the fragmentation mode was higher-energy collisional dissociation (HCD), the normalized collision energy (NCE) was 32, the data-dependent MS/MS was set to Top 20, and the dynamic exclusion time was 60 s. The LC-MS/MS data were processed and analyzed using MaxQuant software (version 1.6.10). The MS/MS spectra were searched against the UniProt *Apis mellifera* (honeybee) reference proteome database (Proteome ID: UP000005203). The digestion enzyme was set as proteinase K, allowing up to two missed cleavages. Carbamidomethylation of cysteine was set as a fixed modification, while oxidation of methionine and acetylation of protein N-termini were set as variable modifications. The false discovery rate (FDR) for both peptide and protein identification was set to 1%. The peptide quantification was performed using the label-free quantification (LFQ) algorithm in MaxQuant, and abundance values were normalized by the total ion current (TIC).

### 2.8. Scoring Method for De Novo Results

Each selected peptide sequence was assigned three subscores. The first subscore, score A, was derived from the peptide’s abundance in the mass spectrometry (MS) spectrum. The abundance values were normalized using the min-max scaling technique to compute score A. The second subscore, score B, was determined by the confidence level of de novo sequencing, with linear normalization applied to the pNovel confidence score values. The third subscore, score C, was associated with the binding energy between the peptide and ACE. The three subscores were weighted as follows: 40% for score A, 30% for score B, and 30% for score C. The aggregate score for each peptide was calculated by summing the weighted values of the three subscores [22,23].

### 2.9. Synthesis of Peptides

Selected peptides were synthesized by Sangon (Shanghai, China). The purity of the synthesized peptides was determined by HPLC to exceed 95%, and their sequences were verified by analytical HPLC-MS/MS, following the manufacturer’s standard quality control protocols. General principles for RP-HPLC and LC-MS/MS analysis of synthetic peptides are available in the literature [24,25].

### 2.10. ACE Inhibition Kinetics

The ACE inhibitory activity of the most potent peptides at varying concentrations was assessed using different concentrations of the substrate FAPGG. The inhibition pattern was elucidated using Lineweaver–Burk plots. The $V_{max}$ and $K_{m}$ values were determined as the Y-axis and X-axis intercepts of the primary plots, respectively. A secondary plot was then constructed by re-plotting the slopes of the Lineweaver–Burk lines against peptide concentrations, and the $K_{i}$ value was obtained from its X-axis intercept.

### 2.11. Molecular Docking Simulation

The 3D structure of the human ACE–lisinopril complex (PDB code: 1O86) was obtained from the Protein Data Bank (https://www.rcsb.org/). After removing lisinopril and crystallographic water, hydrogen atoms were added to the ACE receptor molecule. Ligand structures for IDFDF, DVNFR, and SFHRL were constructed and energy-minimized before docking simulations. Molecular docking was carried out using AutoDock version 4.2.6. The docking simulations were carried out using the Lamarckian genetic algorithm (LGA). A grid box of 50 × 50 × 50 points with a spacing of 0.375 Å was centered on the active site coordinates (x: 36.99; y: 41.25; z: 43.45). For each docking simulation, a total of 10 independent runs were performed, with an initial population size of 150 and a maximum number of 2.5 × 10^6^ energy evaluations. All other parameters were set to their default values. The best-ranked docking poses of the peptides in the ACE active site were selected based on binding energy values.

### 2.12. Statistical Analysis

All data were analyzed with SPSS software (Ver. 16.0; Chicago, IL, USA) and expressed as mean ± standard errors (*n* ≥ 3). Statistical significance among mean values was determined using one-way ANOVA, followed by Duncan’s multiple range test, with a significance threshold of *p* < 0.05.

## 3. Results

### 3.1. In Silico Prediction of Bioactive Peptides Encrypted in MRJPs

**Figure 1:**
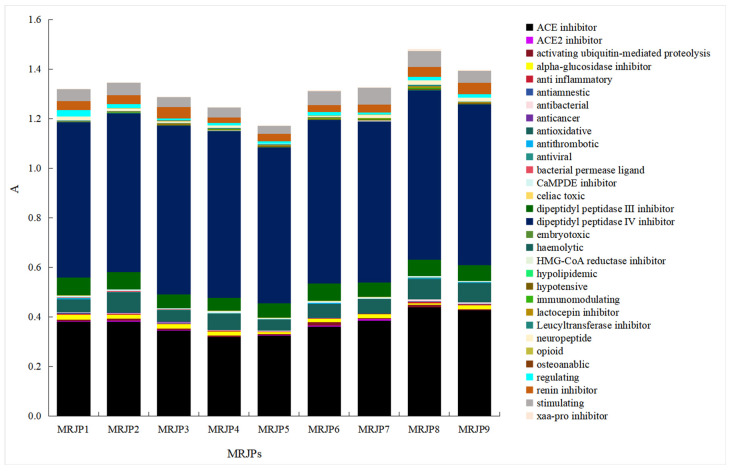
The occurrence frequency of bioactive peptides in MRJPs analyzed in the BIOPEP-UWM database.

Analysis using BIOPEP-UWM revealed that the nine MRJP sequences contained a total of 31 unique bioactive peptide types, as illustrated in Figure 1. Notably, all MRJP sequences shared 14 common bioactive peptide functionalities, including ACE inhibitory activity, activation of ubiquitin-mediated protein hydrolysis, inhibition of alpha-glucosidase, anti-inflammatory properties, antioxidant activity, inhibition of calcium/calmodulin-dependent protein kinase II (CaMPDE), inhibition of dipeptidyl peptidase III (DPP-III), inhibition of DPP-IV, inhibition of lactocepin, neuropeptide regulatory effects, inhibition of renin, stimulatory effects, and inhibition of xaa-pro dipeptidyl peptidase. The cumulative occurrence frequency (∑A) of all peptides within the MRJPs ranged between 1.1738 and 1.4806, with Mrjp5 displaying the lowest and Mrjp8 the highest values.

Among the 31 identified bioactive peptides, dipeptidyl peptidase-IV (DPP-IV) inhibitory peptides and ACE inhibitory peptides exhibited the highest occurrence frequency within each MRJP. The parameter A values for these peptides ranged from 0.6250 to 0.6803 and from 0.3190 to 0.4399, respectively. Specifically, Mrjp8 and Mrjp9 showed the highest parameter A values for ACE inhibitory peptides at 0.4399 and 0.4279, respectively, while Mrjp4 exhibited the lowest A value at 0.3190. These findings suggest that Mrjp8 and Mrjp9 are promising sources for the development of ACE inhibitory peptides.

### 3.2. In Silico Proteolysis of MRJPs for the Production of ACE Inhibitory Peptides

Peptides with specific structures and functionalities are predominantly encoded within protein sequences. Upon release, these fragments can exhibit biological activities. Seven distinct proteases were selected to catalyze the hydrolysis of MRJPs for peptide generation within the BIOPEP-UWM database.

**Figure 2:**
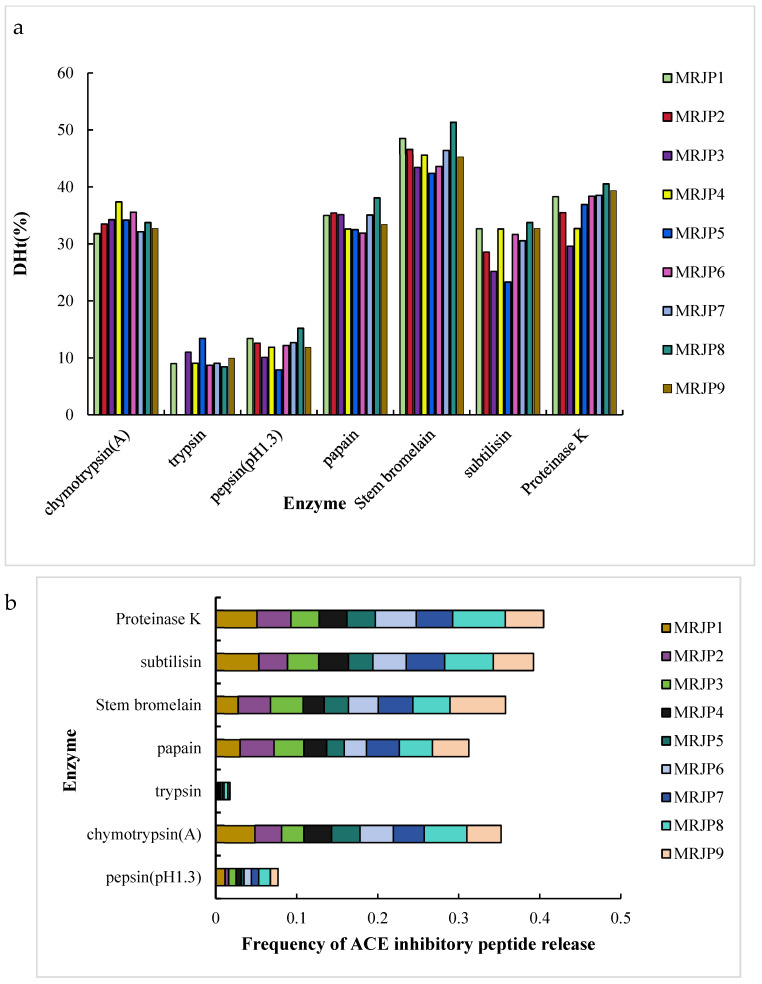
Predicted hydrolysis efficiency (**a**) and ACE-inhibitory-peptide generation frequency (**b**) from virtual proteolysis of MRJPs using distinct enzyme preparations.

The degree of hydrolysis (DHt) of MRJPs achieved using stem bromelain ranged between 43.5780% and 51.3253%, representing the highest observed values (Figure 2a). In contrast, the use of animal enzymes such as trypsin, which yielded a DHt from undetectable levels up to 13.4003%, and pepsin at pH 1.3, which ranged from 7.8727% to 15.1807%, resulted in significantly lower DHt values (Figure 2a). Among the enzymes employed, proteinase K exhibited the highest frequency of release for ACE inhibitory peptides, with a value of 0.4047, followed by subtilisin at 0.3922 (Figure 2b). Trypsin demonstrated the lowest frequency of release for ACE inhibitory peptides, with a value of merely 0.0169 (Figure 2b).

### 3.3. In Vitro Proteolysis of RJ Proteins for the Production of ACE Inhibitory Peptides by RSM

Proteinase K was theoretically identified as the most potent protease for inducing the greatest ACE inhibition activity. However, enzyme–substrate interactions and the extent of hydrolysis observed in silico may not accurately reflect experimental conditions. Therefore, actual proteolysis of RJ proteins was conducted, incorporating insights from in silico hydrolysis results.

In this study, RSM was employed to optimize hydrolysis parameters for producing ACE inhibitory peptides from RJ proteins. Table 2 presents the effects of enzymatic hydrolysis temperature (50–60 °C), pH (8.0–9.0), and hydrolysis time (2–4 h) on the ACE inhibitory activity of the resulting hydrolysates. The outcomes of the variance analysis and the fitness of the model are summarized in Table 3.

The determination coefficient (R^2^) of the model was 0.9868, indicating a strong fit with the actual data and validating its use for predicting variations in ACE inhibitory activity. ANOVA analysis revealed that temperature, pH, and time are statistically significant factors. Both the linear and quadratic components of these factors significantly influenced ACE inhibitory activity, with all *p*-values below 0.01. Additionally, the statistical assessment of the models indicated that the “lack of fit” was not statistically significant (*p* = 0.1072 > 0.05), further substantiating the model’s appropriateness and accuracy.

**Figure 3:**
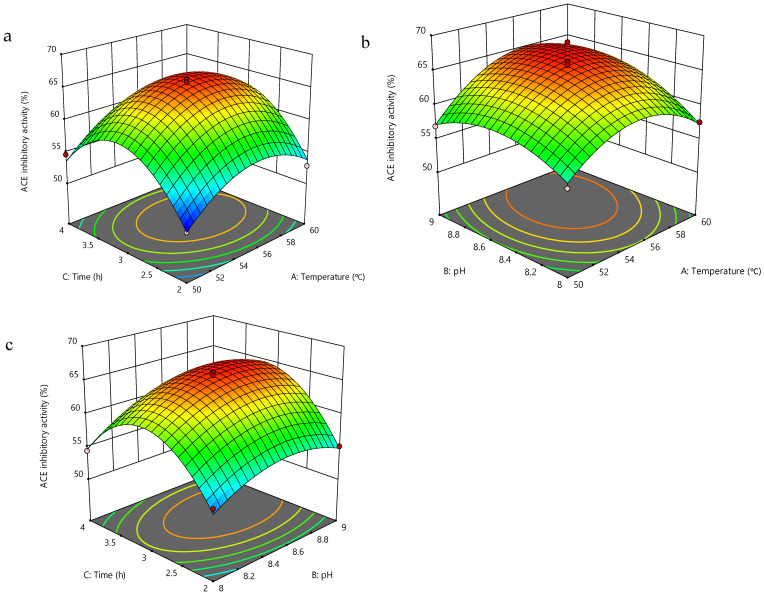
Response surface plots for the effects of independent factors on ACE inhibitory activity of the hydrolysates: (**a**) time and temperature; (**b**) pH and temperature; (**c**) time and pH. Data are presented as mean ± standard deviation (*n* = 3). The color gradient from blue to red represents the ACE inhibitory activity (%), where blue indicates lower activity and red indicates higher activity. The dots represent the experimental design points.

Figure 3 presents the response surface plots to depict the impact of independent variables on the dependent variable. The optimal conditions for achieving maximum ACE inhibitory activity were determined to be a pH of 8.70, a temperature of 56.03 °C, and an enzymolysis duration of 3.09 h. Under these conditions, the yield of the lyophilized hydrolysate was 30.2 ± 1.6% (*w*/*w*, dry basis), and the predicted ACE inhibitory activity of the protein hydrolysates was 66.31%, while the experimental value was 65.58%. This close correlation underscores the reliability of the RSM optimization parameters, making their practical application feasible.

### 3.4. Isolation and Purification of ACE Inhibitory Peptides

**Figure 4:**
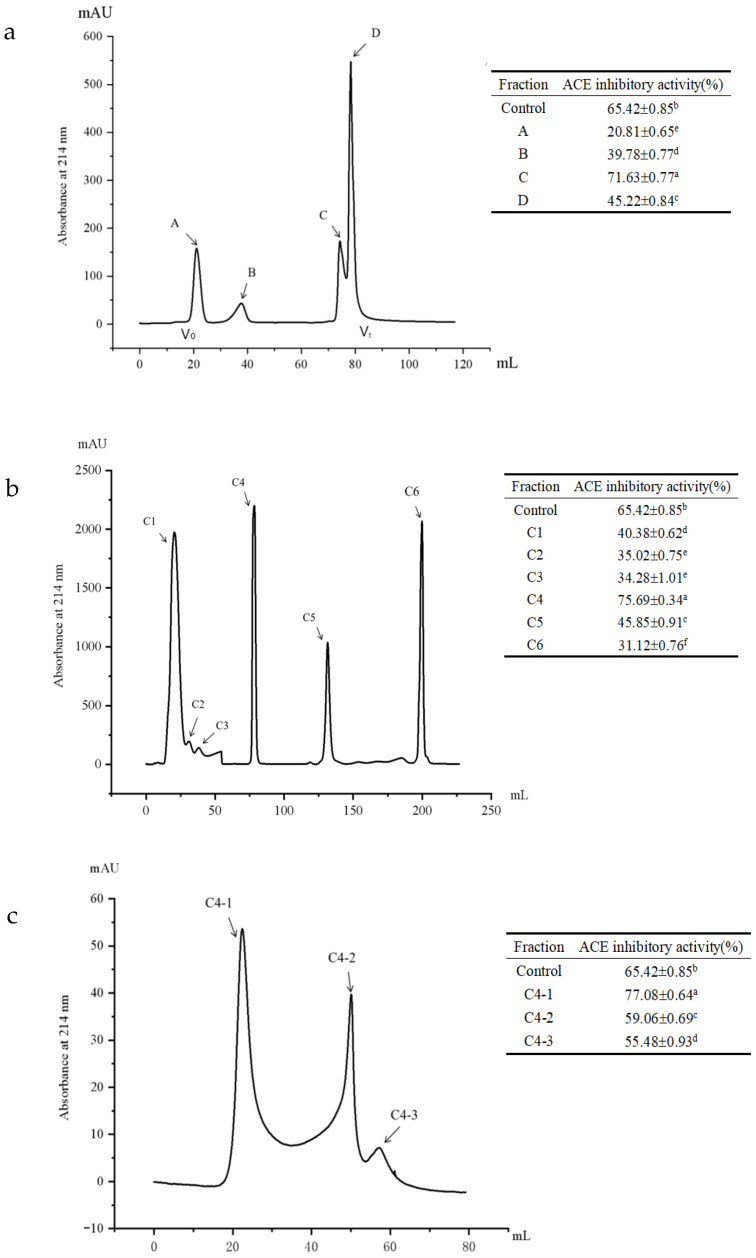
Purification of ACE inhibitory peptides. (**a**) Gel filtration chromatography on Sephadex G-25 of the RJ protein hydrolysate; V_0_: void volume; V_t_: inclusion volume. (**b**) Ion exchange elution chromatography of the C fraction. (**c**) RP-HPLC chromatogram of the C4 fraction. As shown in the inserted table, each fraction was evaluated for ACE inhibitory activity at a uniform concentration of 1.0 mg/mL. Data are presented as mean ± SD (*n* = 3). Different superscript letters (a–f) indicate significant differences between groups (*p* < 0.05), while the same letters indicate no significant difference.

As depicted in Figure 4a, Sephadex G-25 gel column chromatography segregated the hydrolysates into four distinct fractions (A, B, C, and D). Fraction C exhibited significantly higher ACE inhibitory activity compared to the other subfractions (*p* < 0.05), prompting further refinement through anion exchange chromatography. Subsequent purification of fraction C yielded six prominent peaks (C1, C2, C3, C4, C5, and C6) (Figure 4b). Fraction C4, identified as having the highest ACE inhibitory activity, was further purified using RP-HPLC with a semi-preparative C18 column, revealing three major peaks (C4-1, C4-2, and C4-3) (Figure 4c). Fraction C4-1, which exhibited the highest ACE inhibitory activity, was selected for amino acid sequencing using LC-MS/MS.

### 3.5. Identification, Screening and Activities of the ACE Inhibitory Peptides

A total of 57 peptides were identified in C4-1 via mass spectrometry (Table S1). Based on the screening method described in Section 2.8, the top seven peptides by total score were chosen (Table 4). These peptides were KNYPF (Lys-Asn-Tyr-Pro-Phe), VEIPH (Val-Glu-Ile-Pro-His), KPYPDWS (Lys-Pro-Tyr-Pro-Asp-Trp-Ser), IDFDF (Ile-Asp-Phe-Asp-Phe), FDYDFG (Phe-Asp-Tyr-Asp-Phe-Gly), SFHRL (Ser-Phe-His-Arg-Leu), and DVNFR (Asp-Val-Asn-Phe-Arg). The MS/MS spectra of these peptides are shown in Figure S1.

**Figure 5:**
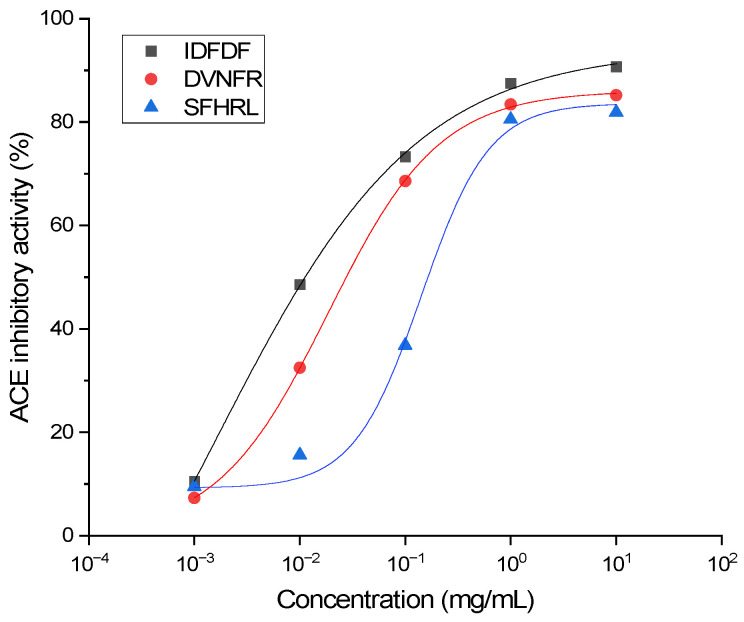
ACE inhibitory activity of chemically synthesized peptides. IC_50_ values (concentration for 50% ACE inhibition) were obtained by fitting the data to a four-parameter logistic function.

Solid-phase synthesis was employed to produce the seven screened peptides, followed by HPLC purification and LC-MS/MS structural verification. The ACE inhibitory activities of each peptide at a concentration of 1 mg/mL were measured in vitro. As summarized in Table 4, the synthesized peptides exhibited ACE inhibitory activities ranging from 61.15 ± 1.89% for FDYDFG to 87.04 ± 1.12% for IDFDF. Among the seven synthesized peptides, IDFDF, DVNFR, and SFHRL exhibited the highest ACE inhibitory activities at 87.04%, 83.26%, and 81.43%, respectively, and were selected for IC_50_ determination. The IC_50_ values for these peptides were 16.9 μM, 42.5 μM, and 242.6 μM, respectively (Figure 5). IDFDF had the lowest IC_50_ value, making it the most effective ACE inhibitor among the synthesized peptides.

### 3.6. Determination of the ACE Inhibition Pattern of the Purified Peptides

**Figure 6:**
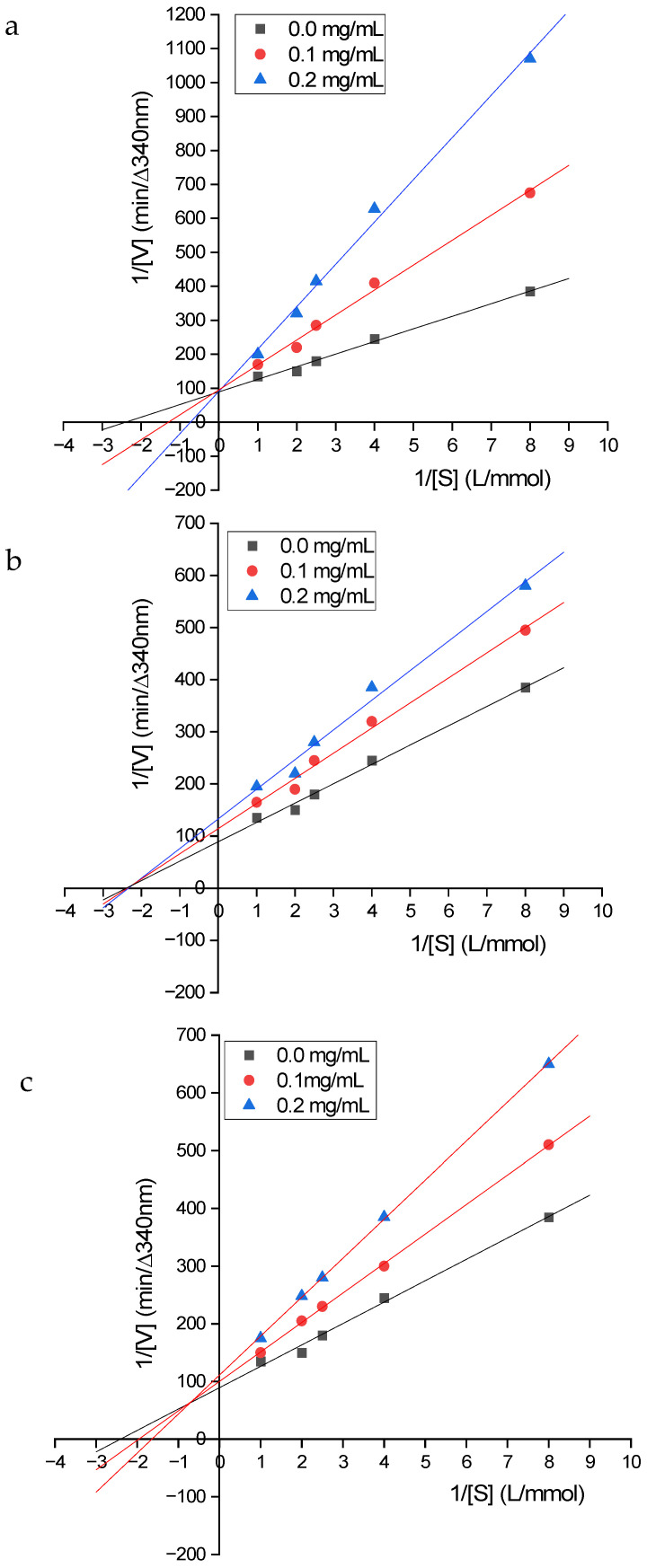
Lineweaver−Burke plot of ACE activity with the peptides (**a**) IDFDF, (**b**) DVNFR, and (**c**) SFHRL.

The ACE inhibition patterns of the three purified peptides with the highest IC_50_ values were assessed using Lineweaver–Burk plots (Figure 6). IDFDF exhibited a competitive inhibition pattern, maintaining a constant $V_{max}$ but varying $K_{m}$ with increasing peptide concentration. DVNFR showed a non-competitive inhibition pattern, with constant $K_{m}$ and varying $V_{max}$ values. SFHRL exhibited a mixed inhibition pattern, as the plots crossed to the left of the 1/V axis but above the 1/S axis.

### 3.7. Molecular Docking

Previous studies have reported that ACE contains a zinc ion (Zn(II)) and three active pockets. The zinc-binding motif includes residues His383, His387, and Glu411. The S1 pocket consists of residues Ala354, Glu384, and Tyr523, while the S2 pocket includes Gln281, His353, Lys511, His513, and Tyr520. The S′ pocket contains only residue Glu162 [17]. Peptides and ACE residues are primarily linked through hydrogen bonds, hydrophobic interactions, and polar, Van der Waals, and electrostatic forces. Hydrogen bond interactions critically contribute to stabilizing the complex architecture and supporting the ACE enzymatic reaction [17].

**Figure 7:**
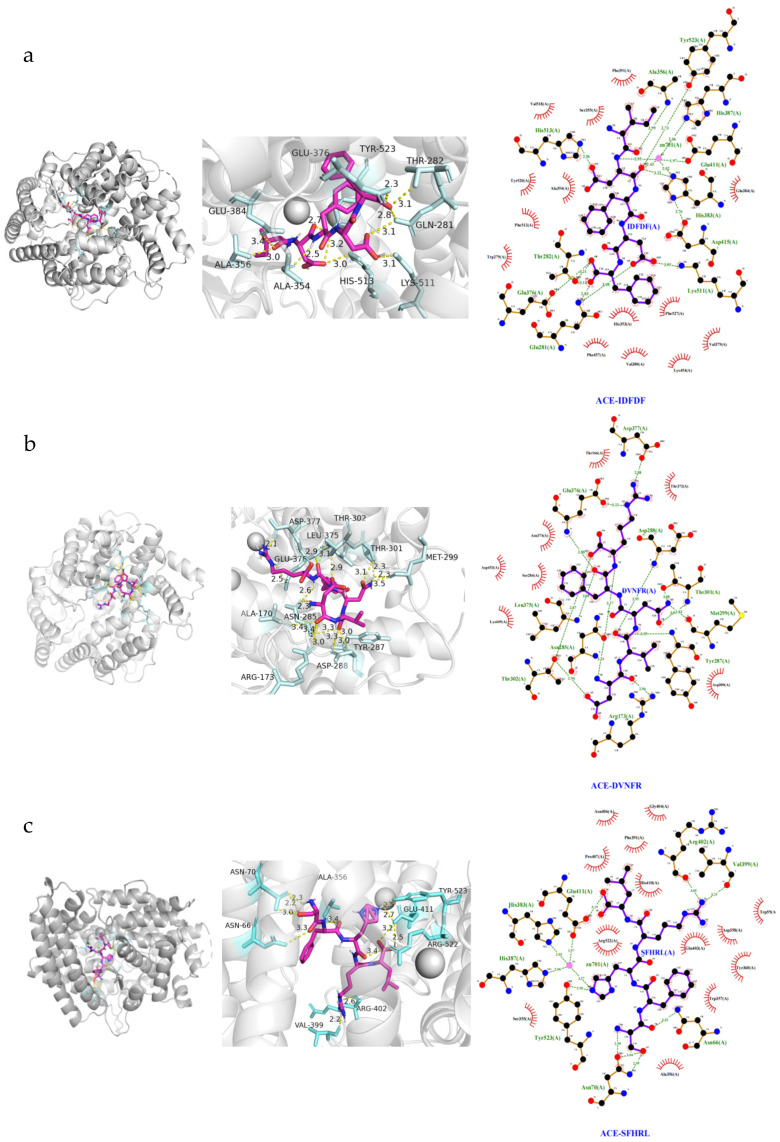
Molecular docking analysis and ligand interaction of (**a**) IDFDF, (**b**) DVNFR, and (**c**) SFHRL with ACE. The pink ball in ACE is a zinc ion.

The binding energies of IDFDF, DVNFR, and SFHRL were determined as −9.5 kcal/mol, −8.9 kcal/mol, and −8.8 kcal/mol, respectively (Table 4). Lower binding energy indicates higher affinity with ACE. The interactions between the three peptides and ACE were evaluated. As shown in Figure 7, IDFDF formed nine hydrogen bonds with Tyr523 (S1 pocket), Gln281 (S2 pocket), Lys511 (S2 pocket), His513 (S2 pocket), His383 (zinc ligand), Ala356, Glu376, and Thr282. Additionally, it coordinated with the Zn(II) of ACE. DVNFR formed the largest number of hydrogen bonds (fourteen) with ACE, but these residues were neither in the ACE active pocket nor in the zinc-ion-binding motif, suggesting non-competitive inhibition. SFHRL formed eight hydrogen bonds with Tyr523 (S1 pocket), Glu411 (zinc ligand), Asn70, Arg402, Val399, and Asn66, and also coordinated with the Zn(II) of ACE, consistent with its mixed inhibition pattern.

## 4. Discussion

Although ACE inhibitory peptides have been identified from MRJP1 through in silico analysis [26] or from RJ proteins via enzymatic hydrolysis [14], this study is the first to generate peptides from nine MRJPs using both in silico and in vitro strategies. While computer-aided simulations can optimize the production of ACE inhibitory peptides, certain constraints of in silico analyses must be acknowledged. In silico proteolysis does not guarantee that computationally generated peptides can be replicated experimentally, as proteins may not be entirely susceptible to protease hydrolysis [27]. Additionally, in silico proteolysis relies on documented cleavage preferences of proteases, which may not always conform to described patterns [28]. The actual proteolytic activity of enzymes is more complex than represented in in silico models. Furthermore, RJ contains other proteins such as aspirin, jelly, and royalsin, as well as peptides that can affect protein hydrolysis results [29]. Therefore, further validation studies on protein hydrolysis in vitro are necessary.

The in silico analysis indicates that RJ proteins are a rich source of bioactive peptides, with ∑A values ranging between 1.1738 and 1.4806, exceeding those reported for quinoa protein (1.0139–1.2508) and soy protein (1.1628–1.2100) [30]. The A values for ACE inhibition in RJ proteins range from 0.3190 to 0.4399, compared to 0.3451–0.4208 for quinoa protein and 0.3833–0.3934 for soy protein [30], suggesting that RJ proteins are a viable source of ACE inhibitory peptides.

In silico enzymatic hydrolysis greatly reduces the workload of enzyme screening. Here, seven proteases from different sources were compared. Plant- and microbial-derived proteases (e.g., stem bromelain, papain, and proteinase K) have broad or no cleavage specificity, leading to higher DHt. In contrast, animal-derived proteases (pepsin and trypsin) have restricted cleavage sites, resulting in lower DHt [31]. Additionally, the in silico simulation for pepsin was performed at pH 1.3, deviating from its optimum (pH 1.5–2.0), which further lowers its DHt and ACE-inhibitory-peptide release. Notably, although stem bromelain had the highest DHt, proteinase K generated the most ACE inhibitory peptides, indicating that hydrolysis efficiency and ACE-inhibitory-peptide release are not directly correlated. Insufficient hydrolysis may fail to release active peptides, while excessive hydrolysis may destroy their activity.

An interesting finding of this study is that the broad-spectrum protease proteinase K was more effective at generating ACE inhibitory peptides than more specific enzymes such as trypsin and chymotrypsin, despite the latter having well-defined and predictable cleavage patterns. Several factors may explain this observation. First, while trypsin (cleaving after K/R) and chymotrypsin (cleaving after F/Y/W) produce predictable fragments, their precise specificity may actually limit the diversity of released peptides, potentially leaving many bioactive motifs embedded within larger, inactive fragments. In contrast, proteinase K, which exhibits broad activity toward hydrophobic and aromatic residues [32], generates a more heterogeneous and generally shorter peptide population, increasing the likelihood of releasing potent ACE inhibitory sequences. Second, as shown in Figure 2b, although trypsin and chymotrypsin achieved reasonable theoretical degrees of hydrolysis (DHt), their predicted frequency of releasing ACE inhibitory peptides was substantially lower than that of proteinase K. This indicates that the quantity of hydrolysis (DHt) does not directly translate to the quality (specific ACE inhibitory activity) of the hydrolysate. This phenomenon has been observed in other food protein systems, where broader-spectrum proteases often outperform more specific ones for bioactive peptide production [27,30].

The ACE inhibitory activity of RJ protein hydrolysates produced using proteinase K was 65.58% compared to 20.65% for whole RJ protein concentrates. This difference may be attributed to the liberation of latent ACE inhibitory peptides through enzyme-catalyzed hydrolysis [33]. Hydrolysis of RJ proteins followed by Sephadex G-25 gel chromatography, ion exchange chromatography, and reverse C18 chromatography further enhanced ACE inhibitory activity. However, the study did not isolate a single active peptide component, possibly due to the semi-preparative C18 chromatographic column’s limited separation efficiency. Higher separation efficiency columns are needed to isolate individual peptide components, though this would reduce separation flux and increase costs.

Identifying peptides with potent ACE inhibitory effects from a vast array of candidates is challenging. Synthesizing and evaluating each peptide’s ACE inhibitory activity is impractical, and predictions based solely on peptide–ACE affinity are highly inaccurate. In RJ protein hydrolysates, the peptide pivotal to ACE inhibitory activity may not be abundant but could have a potent inhibitory effect, while some peptides with moderate inhibitory effects may occur in substantial quantities. This study employed a holistic scoring approach to identify potential ACE inhibitory peptides, as previously used by Sheng et al. [22] for selecting antioxidative peptides from defatted walnut meal hydrolysate and by Zhou et al. [23] for selecting α-amylase inhibitory peptides from quinoa protein hydrolysate.

According to Panyayai et al. [34], peptides with IC_50_ values below 1000 µM are considered promising for blood pressure-lowering effects. The three novel ACE inhibitory peptides identified in this study meet this criterion, with IC_50_ values ranging from 16.9 μM to 242.6 μM. The most potent peptide, IDFDF (IC_50_ = 16.9 μM), is several orders of magnitude less potent than the synthetic drug captopril (0.023 μM) [35], as expected.

Several ACE inhibitory peptides derived from royal jelly proteins have been previously reported, including Ile-Tyr, Val-Tyr, and Lys-Phe, with reported IC_50_ values of 27.18 μM, 71.35 μM, and 16 μM, respectively [14,15]. Compared with these values, IDFDF exhibits comparable or superior activity, particularly relative to Val-Tyr and Ile-Tyr. Furthermore, IDFDF demonstrates excellent activity when compared with other food-derived ACE inhibitors, being comparable to VPP from milk (9 μM) and superior to IKW from egg white (78 μM) [36,37]. These results indicate that IDFDF is a highly potent natural ACE inhibitor, making it a promising candidate for functional food applications.

It should be noted, however, that direct comparisons of IC_50_ values across different studies must be made with caution. The same peptide sequence can exhibit substantially different IC_50_ values depending on experimental conditions, including the source of ACE (e.g., rabbit lung vs. other tissues), substrate concentration (e.g., Hip-His-Leu) and buffer composition, peptide purity or origin (synthetic vs. enzymatic hydrolysate), and methodological differences in IC_50_ calculation. Therefore, while IDFDF clearly demonstrates strong ACE inhibitory activity, comparative interpretations should consider these inherent variabilities among studies.

The in vitro ACE inhibitory assays in this study were performed using ACE isolated from rabbit lungs, while the molecular docking simulations were conducted using the crystal structure of human ACE (PDB code: 1O86). Although rabbit and human ACE share high sequence homology (approximately 85–90% within the catalytic motif) and rabbit ACE is widely accepted as a standard enzyme for such screening due to its commercial availability and similarity to the human enzyme [38], subtle species-specific differences in amino acid residues within the active site may influence peptide binding affinity. Therefore, future validation using recombinant human ACE or human tissue extracts is warranted to confirm the translational relevance of our findings.

IDFDF functions as a competitive inhibitor by binding to ACE’s substrate-binding site, directly impeding substrate access and altering enzymatic activity. DVNFR acts as a non-competitive inhibitor, attaching to a distinct binding site on the enzyme molecule, suppressing ACE activity. SFHRL is a mixed inhibitor, interacting with either the active site or allosteric sites on the enzyme to diminish catalytic efficiency. Notably, no identical peptides have been reported in the BIOPEP-UWM database (http://www.uwm.edu.pl (accessed on 20 March 2025)), indicating that these three ACE inhibitory peptides are novel (Figure S2).

Structurally, all three peptides are pentapeptides characterized by a high content of hydrophobic amino acids (Phe, Ile, and Leu) and charged residues (Asp, Arg, and His). Such sequences are known to resist hydrolysis by pepsin and trypsin, the primary digestive enzymes in the stomach and small intestine [39]. Furthermore, peptides consisting of 2–6 amino acids can be absorbed intact via the peptide transporter PepT1 on intestinal epithelial cells [40]. The five-amino-acid length of these peptides therefore makes them suitable for PepT1-mediated absorption, which is a critical advantage for oral bioavailability.

Collectively, these findings suggest that the three novel ACE inhibitory peptides not only exhibit potent and distinct inhibitory mechanisms but also possess favorable structural features for gastrointestinal stability and intestinal absorption, highlighting their potential as functional food ingredients or lead compounds for antihypertensive therapy.

## 5. Conclusions

In summary, this study revealed that RJ proteins have significant potential for producing various bioactive peptides, particularly DPP-IV and ACE inhibitory peptides. In silico methods for selecting proteases and using RSM to optimize the enzymatic hydrolysis process can efficiently produce high ACE inhibitory hydrolysates. Novel ACE inhibitory peptides, including IDFDF, DVNFR, and SFHRL, were identified through a series of chromatographic separations, screening, and in vitro tests. These peptides exhibited strong ACE inhibitory activity, with IC_50_ values of 16.9 μM, 42.5 μM, and 242.6 μM, respectively. The structure–activity relationships of the peptides were explored using Lineweaver–Burk plots and molecular docking. IDFDF is a competitive inhibitor, DVNFR is a non-competitive inhibitor, and SFHRL is a mixed-type inhibitor. Molecular docking revealed that steric hindrance significantly affects the inhibition mechanism. This study provides new insights into the utilization of RJ proteins and identifies three promising ACE inhibitory peptides. Future work will focus on the antihypertensive effects and in vivo mechanisms of these peptides.

## Figures and Tables

**Table 1:** Experimental variables and coded levels for response surface methodology.

Coded Level	Independent Variable		
	A: Temperature (°C)	B: pH	C: Time (h)
−1	50	8	2
0	55	8.5	3
1	60	9	4

**Table 2:** Design and results of ACE inhibitory activity (%) for the optimization of the hydrolysis conditions by RSM. Data are presented as mean ± standard deviation (*n* = 3).

Number	A: Temperature (°C)	B: pH	C: Time (h)	ACE Inhibitory Activity (%)
1	50	8	3	55.62 ± 0.88
2	60	8	3	57.54 ± 0.79
3	50	9	3	56.93 ± 1.20
4	60	9	3	63.89 ± 0.95
5	50	8.5	2	50.80 ± 1.10
6	60	8.5	2	52.78 ± 0.87
7	50	8.5	4	54.60 ± 0.89
8	60	8.5	4	57.51 ± 0.69
9	55	8	2	53.71 ± 0.93
10	55	9	2	55.10 ± 0.73
11	55	8	4	54.45 ± 0.88
12	55	9	4	58.82 ± 1.13
13	55	8.5	3	64.89 ± 1.04
14	55	8.5	3	66.30 ± 0.85
15	55	8.5	3	65.41 ± 0.96
16	55	8.5	3	66.20 ± 1.22
17	55	8.5	3	65.89 ± 1.51

**Table 3:** Analysis of variance of the regression coefficients of the fitted equation for ACE inhibitory activity.

Source	Sum of Squares	df	Mean Square	F-Value	*p*-Value Prob > F
Model	453.42	9	50.38	58.08	<0.0001 ^a^
A-Temp	23.81	1	23.81	27.44	0.0012 ^a^
B-pH	22.78	1	22.78	26.26	0.0014 ^a^
C-TM	20.8	1	20.80	23.98	0.0018 ^a^
AB	6.5	1	6.5	7.5	0.0290 ^a^
AC	0.2025	1	0.2025	0.2334	0.6437
BC	2.25	1	2.25	2.59	0.1513
A2	81.52	1	81.52	93.97	<0.0001 ^a^
B2	33.6	1	33.6	38.74	0.0004 ^a^
C2	229.01	1	229.01	263.99	<0.0001 ^a^
Residual	6.07	7	0.8675		
Lack of Fit	4.55	3	1.52	3.99	0.1072
Pure error	1.52	4	0.38		
Cor Totol	459.50	16			
Std.Dev.	0.93	R-Squared	0.9868		
Mean	73.83	Adj R-Squared	0.9698		
C.V.%	1.26	Pred R-Squared	0.8363		
PRESS	75.22	Adeq Precision	20.841		

^a^ Significant within a 99% confidence interval.

**Table 4:** The scoring and ACE inhibitory activity of the selected ACE inhibitory peptides.

Peptide Sequence	Abundance	Score A	Confidence	Score B	Binding Energy	Score C	Total Score	ACE Inhibitory Activity (%)
KNYPF	1.58 × 10^10^	95.05	418.0	99.09	−8.4	88.42	94.27	78.56 ± 1.24
VEIPH	1.41 × 10^10^	84.53	420.6	99.72	−8.6	90.53	90.89	75.78 ± 1.02
KPYPDWS	1.61 × 10^10^	96.26	420.2	99.62	−7.7	81.05	92.71	71.55 ± 1.91
IDFDF	1.50 × 10^10^	90.02	416.2	98.67	−9.5	100.00	95.61	87.04 ± 1.12
FDYDFG	1.60 × 10^10^	95.82	412.5	97.80	−6.8	71.58	89.14	61.15 ± 1.89
SFHRL	1.67 × 10^10^	100.00	421.8	100.0	−8.8	92.63	97.79	81.43 ± 1.25
DVNFR	1.56 × 10^10^	93.41	419.8	99.53	−8.9	93.68	95.32	83.26 ± 1.37

## Data Availability

The original contributions presented in this study are included in the article/Supplementary Materials. Further inquiries can be directed to the corresponding author.

## Supplementary Materials

The following supporting information can be downloaded at https://www.mdpi.com/article/10.3390/foods15091536/s1, Figure S1: Seven screened peptides from RJ proteins identified by LC-MS/MS. Note: MS/MS spectra of (a) KNYPF, (b) VEIPH, (c) KPYPDWS, (d) IDFDF, (e) FDYDFG, (f) SFHRL, and (g) DVNFR; Figure S2: Search process and results for the three peptides (IDFDF, DVNFR, and SFHRL) in the BIOPEP-UWM database; Table S1: 57 peptides were identified in C4-1 via mass spectrometry.
