# Supplementary material for: In Silico and In Vitro Screening and Mechanisms of Angiotensin I-Converting Enzyme Inhibitory Peptides from Protein Hydrolysates of Royal Jelly
Source: Foods. 2026 Apr 29;15(9):1536. doi: 10.3390/foods15091536 (PMC13164353; doi:10.3390/foods15091536)
Supplement: Supplementary file 1 [file foods-15-01536-s001.zip › foods-4232823-supplementary/Supporting_Information.pdf]

Supporting Information

In silico and in vitro screening and mechanisms of angiotensin I-converting enzyme inhibitory peptides from protein hydrolysates of royal jelly

Ying Zhang[1], Shipeng Guo [2], Haoxiang Miao [2], Yafei Gu [2], and Jian Zhang [2,*]

[1] College of Food Science and Engineering, Tianjin University of Science and Technology, Tianjin 300457, China

[2] College of Biotechnology, Tianjin University of Science and Technology, Tianjin 300457, China

* Corresponding author: Jian Zhang

E-mail: zj96sk@tust.edu.cn

Tel: +86-022-60602715

Present address: No. 29, 13th Avenue, TEDA Tianjin 300457, P. R. China

Contents

Figure S1. Seven screened peptides from RJ proteins identified by LC-MS/MS. Note: MS/MS spectra of (a)KNYPF, (b)VEIPH, (c)KPYPDWS,(d) IDFDF,(e) FDYDFG, (f)SFHRL, and (g)DVNFR

Figure S2. Search process and results for the three peptides (IDFDF, DVNFR, and SFHRL) in the BIOPEP-UWM database.

Table S1. 57 peptides were identified in C4-1 via mass spectrometry

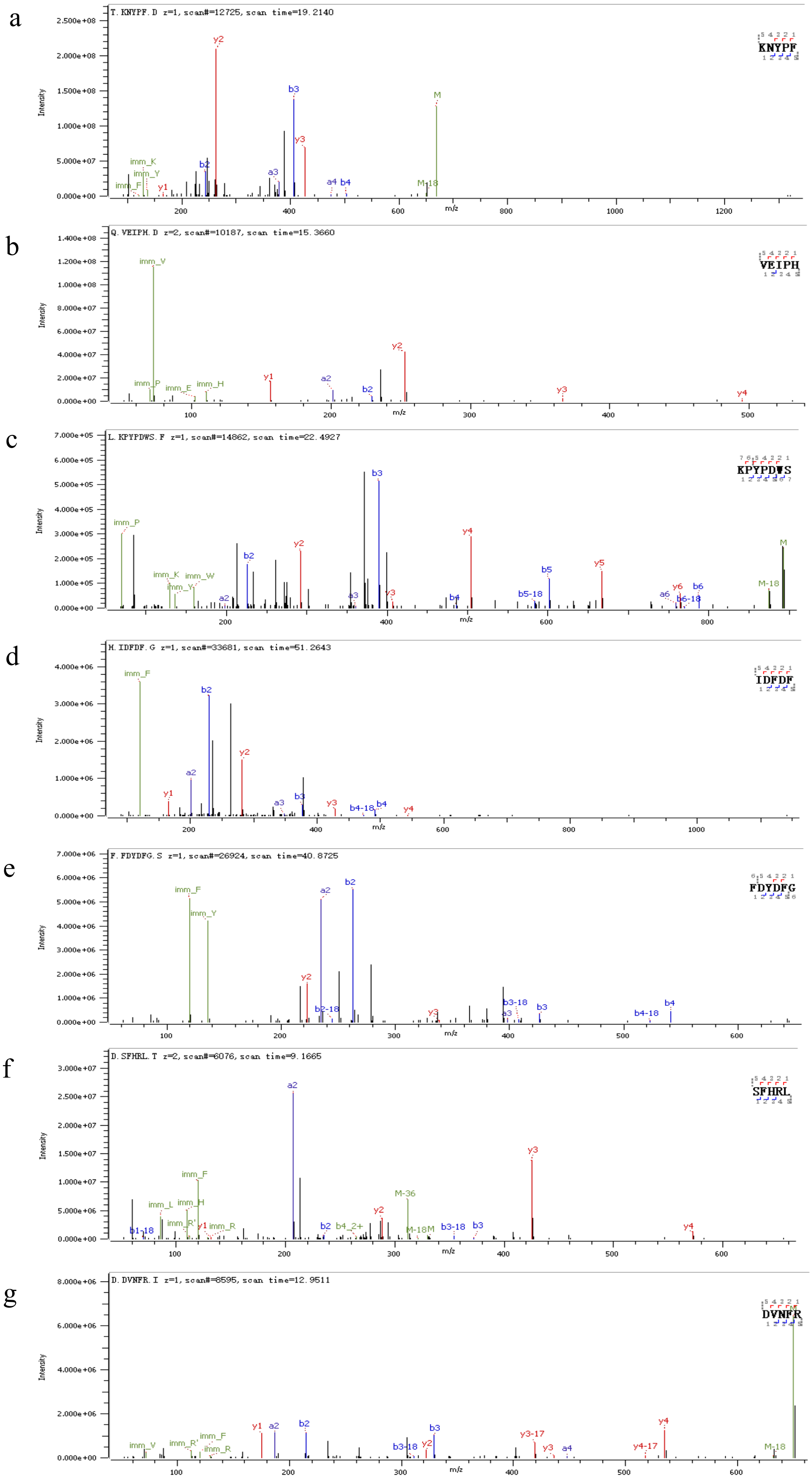
a
T.KNYPF.D z=1, scan#=12725, scan time=19.2140
KNYPF
b
Q.VEIPH.D z=2, scan#=10187, scan time=15.3660
VEIPH
c
L.KPYPDWS.F z=1, scan#=14862, scan time=22.4927
KPYPDWS
d
H.IDFDF.G z=1, scan#=33681, scan time=51.2643
IDFDF
e
F.FDYDFG.S z=1, scan#=26924, scan time=40.8725
FDYDFG
f
D.SFHRL.T z=2, scan#=6076, scan time=9.1665
SFHRL
g
D.DVNFR.I z=1, scan#=8595, scan time=12.9511
DVNFR
Intensity
m/z

Figure S1

1. IDFDF

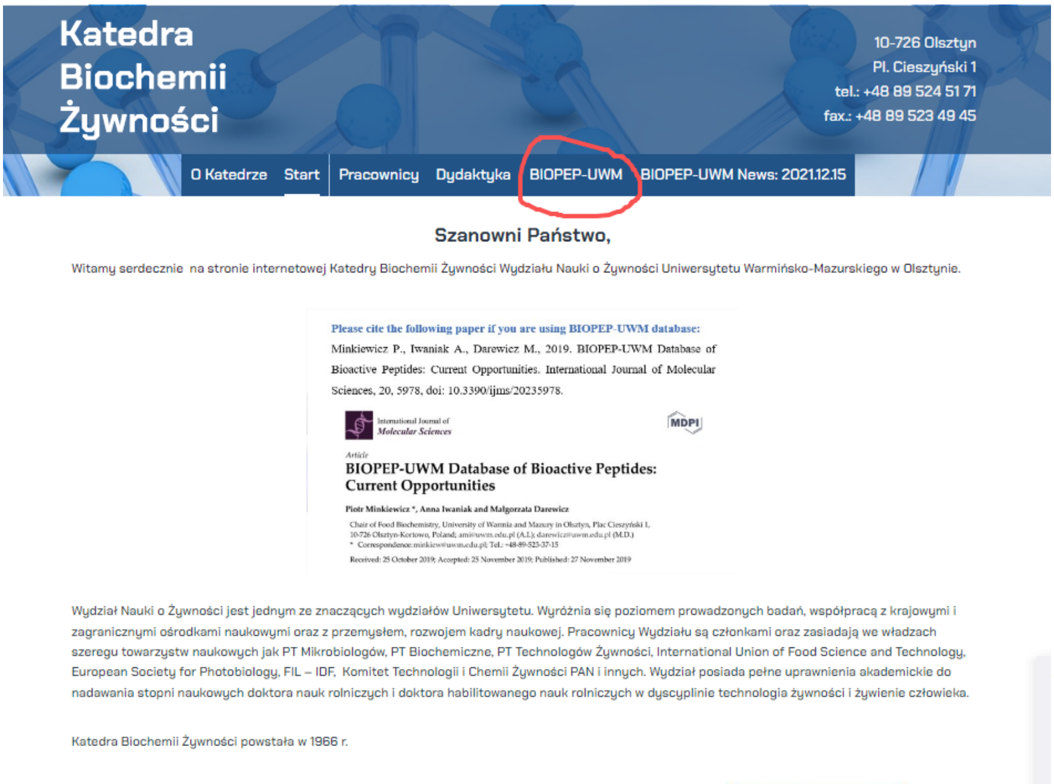
Katedra
Biochemii
Żywności
10-726 Olsztyn
Pl. Cieszyński 1
tel.: +48 89 524 51 71
fax.: +48 89 523 49 45
O Katedrze
Start
Pracownicy
Dydaktyka
BIOPEP-UWM
BIOPEP-UWM News: 2021.12.15
Szanowni Państwo,
Witamy serdecznie na stronie internetowej Katedry Biochemii Żywności Wydziału Nauki o Żywności Uniwersytetu Warmińsko-Mazurskiego w Olsztynie.
Please cite the following paper if you are using BIOPEP-UWM database:
Minkiewicz P., Iwaniak A., Darewicz M., 2019. BIOPEP-UWM Database of Bioactive Peptides: Current Opportunities. International Journal of Molecular Sciences, 20, 5978, doi: 10.3390/ijms/20235978.
International Journal of Molecular Sciences
MDPI
Article
BIOPEP-UWM Database of Bioactive Peptides: Current Opportunities
Piotr Minkiewicz *, Anna Iwaniak and Małgorzata Darewicz
Wydział Nauki o Żywności jest jednym ze znaczących wydziałów Uniwersytetu. Wyróżnia się poziomem prowadzonych badań, współpracą z krajowymi i zagranicznymi ośrodkami naukowymi oraz z przemysłem, rozwojem kadry naukowej. Pracownicy Wydziału są członkami oraz zasiadają we władzach szeregu towarzystw naukowych jak PT Mikrobiologów, PT Biochemiczne, PT Technologów Żywności, International Union of Food Science and Technology, European Society for Photobiology, FIL – IDF, Komitet Technologii i Chemii Żywności PAN i innych. Wydział posiada pełne uprawnienia akademickie do nadawania stopni naukowych doktora nauk rolniczych i doktora habilitowanego nauk rolniczych w dyscyplinie technologia żywności i żywienie człowieka.
Katedra Biochemii Żywności powstała w 1966 r.

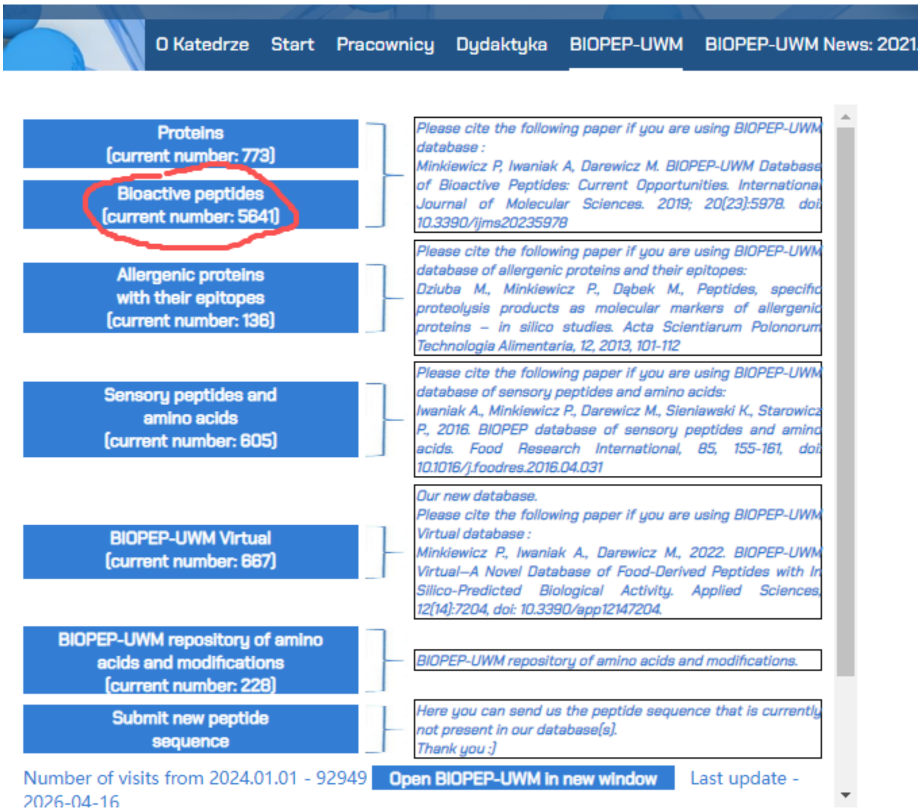
O Katedrze
Start
Pracownicy
Dydaktyka
BIOPEP-UWM
BIOPEP-UWM News: 2021
Proteins
(current number: 773)
Bioactive peptides
(current number: 5641)
Please cite the following paper if you are using BIOPEP-UWM database :
Minkiewicz P, Iwaniak A, Darewicz M. BIOPEP-UWM Database of Bioactive Peptides: Current Opportunities. International Journal of Molecular Sciences. 2019; 20(23):5978. doi 10.3390/ijms20235978
Allergenic proteins with their epitopes
(current number: 136)
Please cite the following paper if you are using BIOPEP-UWM database of allergenic proteins and their epitopes:
Dziuba M., Minkiewicz P., Dąbek M., Peptides, specific proteolysis products as molecular markers of allergenic proteins – in silico studies. Acta Scientiarum Polonorum Technologia Alimentaria, 12, 2013, 101-112
Sensory peptides and amino acids
(current number: 605)
Please cite the following paper if you are using BIOPEP-UWM database of sensory peptides and amino acids:
Iwaniak A., Minkiewicz P., Darewicz M., Sieniawski K., Starowicz P., 2016. BIOPEP database of sensory peptides and amino acids. Food Research International, 85, 155-161, doi 10.1016/j.foodres.2016.04.031
BIOPEP-UWM Virtual
(current number: 667)
Our new database.
Please cite the following paper if you are using BIOPEP-UWM Virtual database :
Minkiewicz P., Iwaniak A., Darewicz M., 2022. BIOPEP-UWM Virtual—A Novel Database of Food-Derived Peptides with In Silico-Predicted Biological Activity. Applied Sciences, 12(14):7204, doi: 10.3390/app12147204.
BIOPEP-UWM repository of amino acids and modifications
(current number: 228)
BIOPEP-UWM repository of amino acids and modifications.
Submit new peptide sequence
Here you can send us the peptide sequence that is currently not present in our database(s).
Thank you :)
Number of visits from 2024.01.01 - 92949
Open BIOPEP-UWM in new window
Last update - 2026-04-16

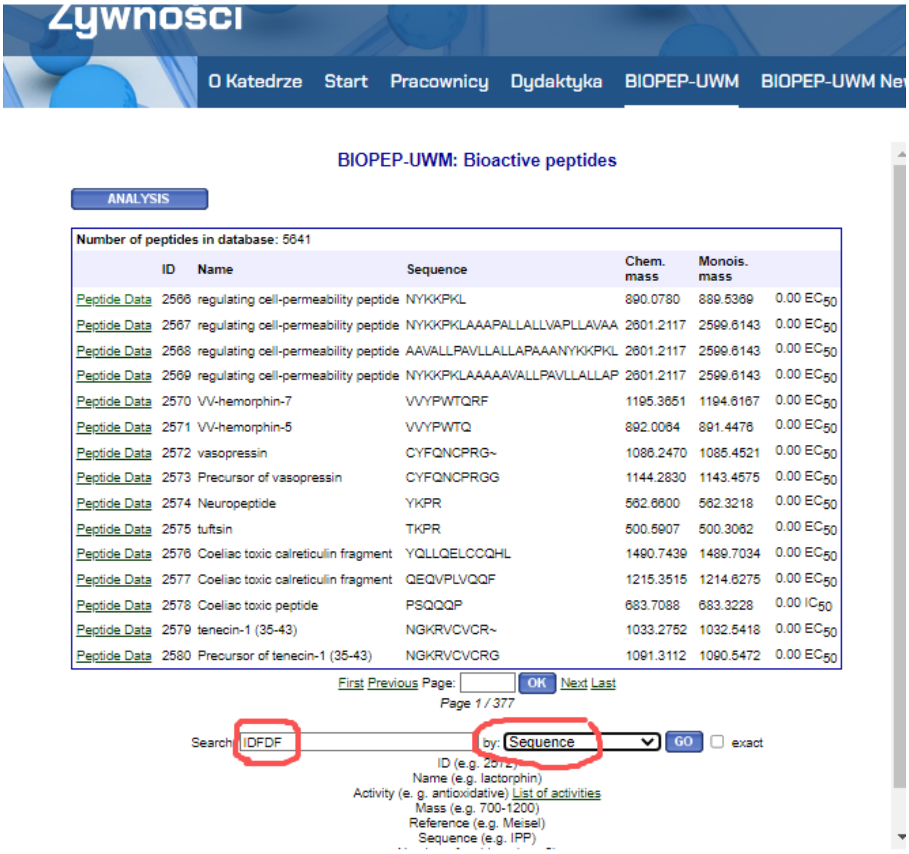

Żywności

O Katedrze Start Pracownicy Dydaktyka BIOPEP-UWM BIOPEP-UWM Ne

BIOPEP-UWM: Bioactive peptides

ANALYSIS

Number of peptides in database: 5641

	ID	Name	Sequence	Chem. mass	Monois. mass	
Peptide Data	2566	regulating cell-permeability peptide	NYKKPKL	890.0780	889.5369	0.00 EC_{50}
Peptide Data	2567	regulating cell-permeability peptide	NYKKPKLAAAPALLALLVAPLLAVAA	2601.2117	2599.6143	0.00 EC_{50}
Peptide Data	2568	regulating cell-permeability peptide	AAVALLPAVLLALLAPAAANYKKPKL	2601.2117	2599.6143	0.00 EC_{50}
Peptide Data	2569	regulating cell-permeability peptide	NYKKPKLAAAAAVALLPAVLLALLAP	2601.2117	2599.6143	0.00 EC_{50}
Peptide Data	2570	VV-hemorphin-7	VVYPWTQRF	1195.3651	1194.6167	0.00 EC_{50}
Peptide Data	2571	VV-hemorphin-5	VVYPWTQ	892.0064	891.4476	0.00 EC_{50}
Peptide Data	2572	vasopressin	CYFQNCPRG~	1086.2470	1085.4521	0.00 EC_{50}
Peptide Data	2573	Precursor of vasopressin	CYFQNCPRGG	1144.2830	1143.4575	0.00 EC_{50}
Peptide Data	2574	Neuropeptide	YKPR	562.6600	562.3218	0.00 EC_{50}
Peptide Data	2575	tuftsin	TKPR	500.5907	500.3062	0.00 EC_{50}
Peptide Data	2576	Coeliac toxic calreticulin fragment	YQLLQELCCQHL	1490.7439	1489.7034	0.00 EC_{50}
Peptide Data	2577	Coeliac toxic calreticulin fragment	QEQVPLVQQF	1215.3515	1214.6275	0.00 EC_{50}
Peptide Data	2578	Coeliac toxic peptide	PSQQQP	683.7088	683.3228	0.00 IC_{50}
Peptide Data	2579	tenecin-1 (35-43)	NGKRVCVCR~	1033.2752	1032.5418	0.00 EC_{50}
Peptide Data	2580	Precursor of tenecin-1 (35-43)	NGKRVCVCRG	1091.3112	1090.5472	0.00 EC_{50}

First Previous Page: OK Next Last

Page 1 / 377

Search: IDFDF by: Sequence GO exact

ID (e.g. 2572)
Name (e.g. lactorphin)
Activity (e. g. antioxidative) List of activities
Mass (e.g. 700-1200)
Reference (e.g. Meisel)
Sequence (e.g. IPP)

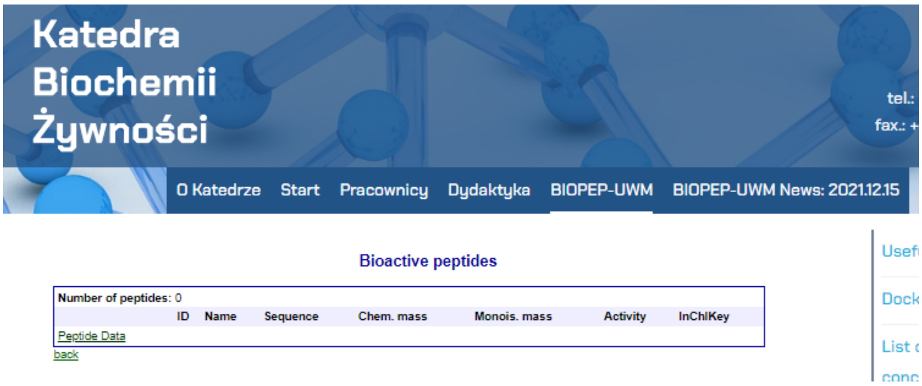

Katedra Biochemii Żywności

tel.:
fax.: +

O Katedrze Start Pracownicy Dydaktyka BIOPEP-UWM BIOPEP-UWM News: 2021.12.15

Bioactive peptides

Number of peptides: 0

	ID	Name	Sequence	Chem. mass	Monois. mass	Activity	InChiKey
Peptide Data							

back

Usef
Dock
List c
conc

2. DVNFR

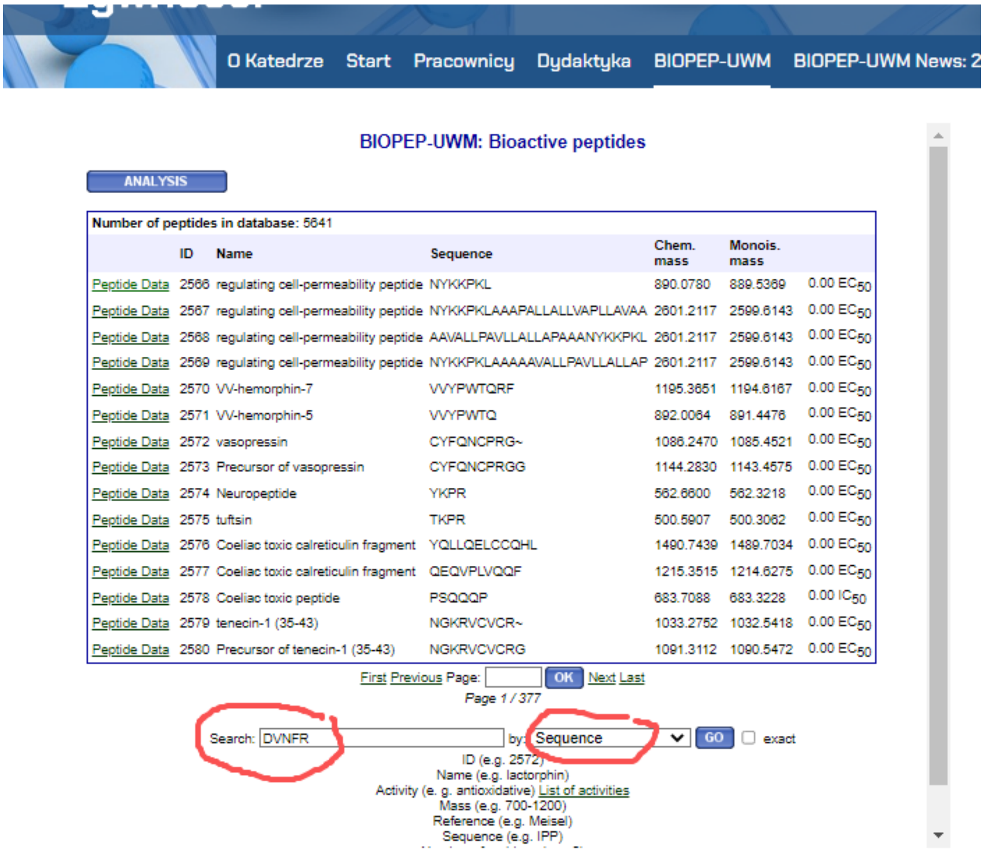

O Katedrze Start Pracownicy Dydaktyka BIOPEP-UWM BIOPEP-UWM News: 2

BIOPEP-UWM: Bioactive peptides

ANALYSIS

Number of peptides in database: 5641

	ID	Name	Sequence	Chem. mass	Monois. mass	
Peptide Data	2566	regulating cell-permeability peptide	NYKKPKL	890.0780	889.5369	0.00 EC_{50}
Peptide Data	2567	regulating cell-permeability peptide	NYKKPKLAAAPALLALLVAPLLAVAA	2601.2117	2599.6143	0.00 EC_{50}
Peptide Data	2568	regulating cell-permeability peptide	AAVALLPAVLLALLAPAAANYKKPKL	2601.2117	2599.6143	0.00 EC_{50}
Peptide Data	2569	regulating cell-permeability peptide	NYKKPKLAAAAAVALLPAVLLALLAP	2601.2117	2599.6143	0.00 EC_{50}
Peptide Data	2570	VV-hemorphin-7	VVYPWTQRF	1195.3651	1194.6167	0.00 EC_{50}
Peptide Data	2571	VV-hemorphin-5	VVYPWTQ	892.0064	891.4476	0.00 EC_{50}
Peptide Data	2572	vasopressin	CYFQNCPRG~	1086.2470	1085.4521	0.00 EC_{50}
Peptide Data	2573	Precursor of vasopressin	CYFQNCPRGG	1144.2830	1143.4575	0.00 EC_{50}
Peptide Data	2574	Neuropeptide	YKPR	562.6600	562.3218	0.00 EC_{50}
Peptide Data	2575	tuftsin	TKPR	500.5907	500.3062	0.00 EC_{50}
Peptide Data	2576	Coeliac toxic calreticulin fragment	YQLLQELCCQHL	1490.7439	1489.7034	0.00 EC_{50}
Peptide Data	2577	Coeliac toxic calreticulin fragment	QEQVPLVQQF	1215.3515	1214.6275	0.00 EC_{50}
Peptide Data	2578	Coeliac toxic peptide	PSQQQP	683.7088	683.3228	0.00 IC_{50}
Peptide Data	2579	tenecin-1 (35-43)	NGKRVCVCR~	1033.2752	1032.5418	0.00 EC_{50}
Peptide Data	2580	Precursor of tenecin-1 (35-43)	NGKRVCVCRG	1091.3112	1090.5472	0.00 EC_{50}

First Previous Page: OK Next Last

Page 1 / 377

Search: DVNFR by: Sequence GO exact

ID (e.g. 2572)
Name (e.g. lactorphin)
Activity (e. g. antioxidative) List of activities
Mass (e.g. 700-1200)
Reference (e.g. Meisel)
Sequence (e.g. IPP)

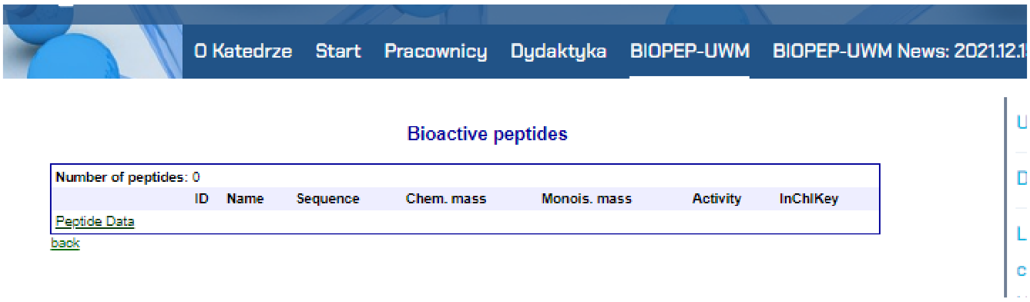

O Katedrze Start Pracownicy Dydaktyka BIOPEP-UWM BIOPEP-UWM News: 2021.12.1

Bioactive peptides

Number of peptides: 0

	ID	Name	Sequence	Chem. mass	Monois. mass	Activity	InChIKey
Peptide Data							

back

3. SFHRL

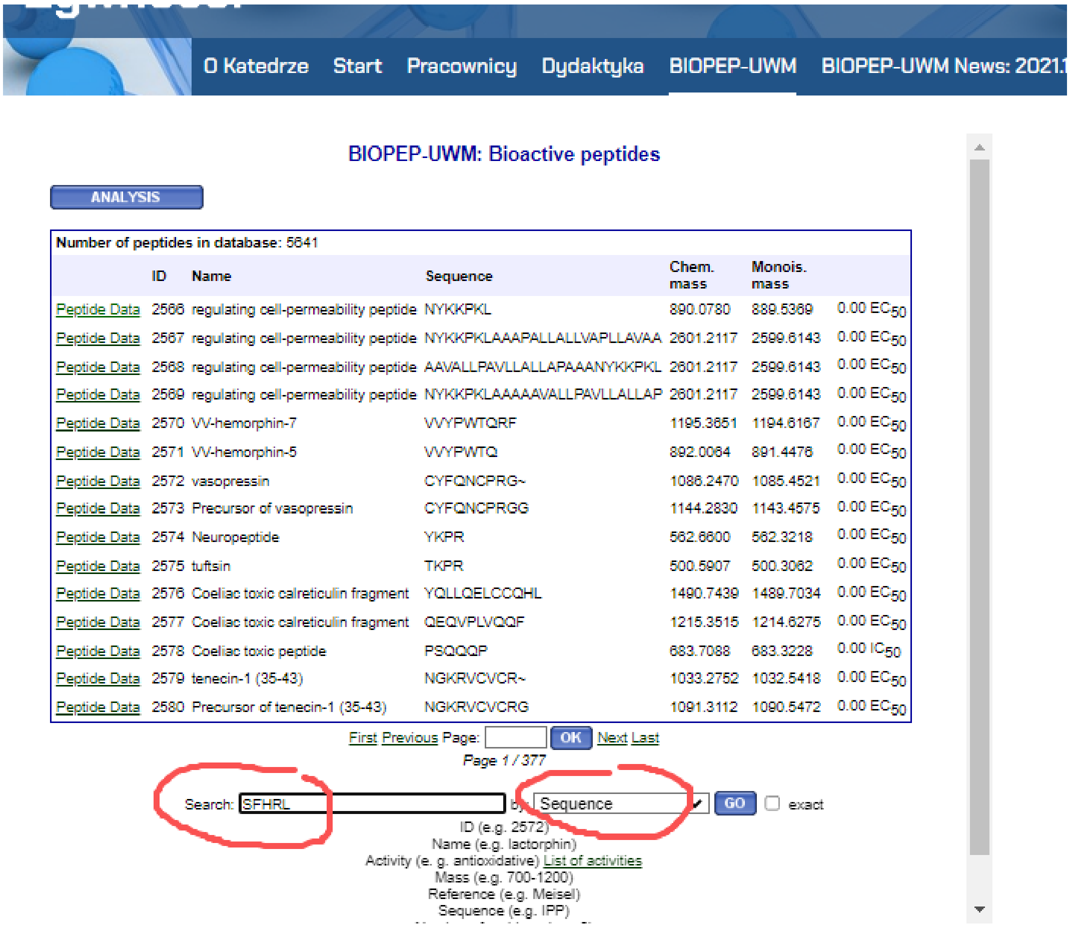

Number of peptides in database: 5641

	ID	Name	Sequence	Chem. mass	Monois. mass	
Peptide Data	2566	regulating cell-permeability peptide	NYKKPKL	890.0780	889.5369	0.00 EC_{50}
Peptide Data	2567	regulating cell-permeability peptide	NYKKPKLAAAPALLALLVAPLLAVAA	2601.2117	2599.6143	0.00 EC_{50}
Peptide Data	2568	regulating cell-permeability peptide	AAVALLPAVLLALLAPAAANYKKPKL	2601.2117	2599.6143	0.00 EC_{50}
Peptide Data	2569	regulating cell-permeability peptide	NYKKPKLAAAAAVALLPAVLLALLAP	2601.2117	2599.6143	0.00 EC_{50}
Peptide Data	2570	VV-hemorphin-7	VVYPWTQRF	1195.3651	1194.6167	0.00 EC_{50}
Peptide Data	2571	VV-hemorphin-5	VVYPWTQ	892.0064	891.4476	0.00 EC_{50}
Peptide Data	2572	vasopressin	CYFQNCPRG~	1086.2470	1085.4521	0.00 EC_{50}
Peptide Data	2573	Precursor of vasopressin	CYFQNCPRGG	1144.2830	1143.4575	0.00 EC_{50}
Peptide Data	2574	Neuropeptide	YKPR	562.6600	562.3218	0.00 EC_{50}
Peptide Data	2575	tuftsin	TKPR	500.5907	500.3062	0.00 EC_{50}
Peptide Data	2576	Coeliac toxic calreticulin fragment	YQLLQELCCQHL	1490.7439	1489.7034	0.00 EC_{50}
Peptide Data	2577	Coeliac toxic calreticulin fragment	QEQVPLVQQF	1215.3515	1214.6275	0.00 EC_{50}
Peptide Data	2578	Coeliac toxic peptide	PSQQQP	683.7088	683.3228	0.00 IC_{50}
Peptide Data	2579	tenecin-1 (35-43)	NGKRVCVCR~	1033.2752	1032.5418	0.00 EC_{50}
Peptide Data	2580	Precursor of tenecin-1 (35-43)	NGKRVCVCRG	1091.3112	1090.5472	0.00 EC_{50}

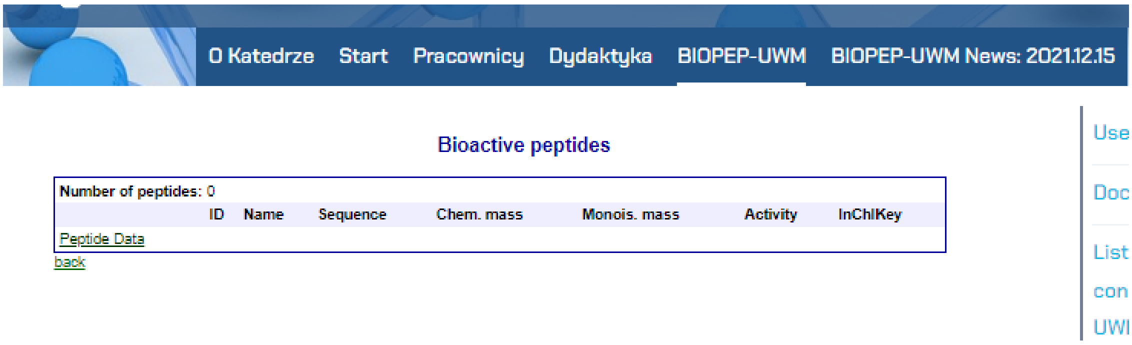

Number of peptides: 0

	ID	Name	Sequence	Chem. mass	Monois. mass	Activity	InChIKey
Peptide Data							

back

Figure S2
